# A Rare Embryologic Variation: Anterior Communicating Artery Aneurysm Associated with Carotid—Anterior Cerebral Artery Anastomosis or Infraoptic Course of the Anterior Cerebral Artery

**DOI:** 10.4137/ccrep.s918

**Published:** 2008-09-24

**Authors:** Alaattin Yurt, Kubilay Uçar, Füsun Özer, İsmail Oran, Nuri Arda

**Affiliations:** 1 Department of Neurosurgery, İzmir Training and Research Hospital, İzmir, Turkey.; 2 Department of Radiology, Ege University School of Medicine, İzmir, Turkey.; 3 Department of Neurosurgery, Dokuz Eylül Üniversity School of Medicine, İzmir, Turkey.

**Keywords:** anomaly, anterior cerebral artery, cerebral aneurysms, cerebral arteries

## Abstract

Aneurysms of the complex of the anterior cerebral artery are frequently associated with anatomic variations of the circle of Willis. We describe a case of aneurysmal rupture of the anterior communicating artery, a variant of the anterior cerebral artery. The aneurysm appeared to be situated on this vessel proximal to the infered site of the AcoA. Surgery was performed at the 6th day after hemorrhage. The anterior communicating artery aneurysm was clipped. The post operative course was unventful, with complete recovery. In our case, an extremely rare variation of the proximal tract of the anterior cerebral artery, i.e. an infraoptic course of the proximal precommunicating tract under the optic nerve, with the distal A1 tract anterior to the chiasm and positioned between the optic nerves, is presented.

## Introduction

The purpose of this presentation is to define the characteristic of a rare anomalous cerebral artery and to discuss some possibilities concerning its origin. Carotid-anterior cerebral artery anastomosis constitutes an anomaly of the anterior part of the arterial circle of the brain (Bassett, 1949; Besson et al. 1980; Bollar et al. 1988; Chioffi et al. 1997; Kessler, 1979; Kwak, 1980; Lasjaunias et al. 2001; Mercier et al. 1989; Yaşargil, 1984). The anterior cerebral a. arises a few millimetres above the emergence of the internal carotid from the cavernous sinus, at the usual level of the ophthalmic a. It travels medially, beneath the optic n., and then describes a curve with a superolateral concavity to arrive at the anterior communicating a. (Lasjaunias et al. 2001; Yaşargil, 1984). This variant is often associated with other cerebral vasculer anomalies, especially arterial aneurysm (Chioffi et al. 1997; Kessler, 1979; Kwak et al. 1980; Lasjaunias et al. 2001).

This anomaly was associated with a ruptured anterior communicating artery (AcomA) aneurysm. It is concluded that, for aneurysm surgery, careful angiographic evaluation and an understanding of the neurovascular relationships in the circle of Willis are essential for a successful postoperative course, especially when very rare vascular anomalies are treated.

This report describes a rare case of anterior communicating artery aneurysm associated with carotid—anterior cerebral artery anastomosis or infraoptic course of the anterior cerebral artery which was treated by surgical approach.

## Case Report

A 35-year-old male patient admitted to our clinic suffering from headaches with vomiting and loss of consciousness. Examination revealed mild confusion, moderate neck stiffness, and early papilledema. Radiological examination: Computed Tomography (CT) revealed subarachnoid hemorrhage in the basal and sylvian cisterns. Angiography (Figs. 1a and 1b) documented an anterior communicating artery aneurysm; the angiogram also demonstrated a low bifurcation of the right internal carotid artery (ICA), filling of the distal part (A2) of both anterior cerebral arteries from an anomalous A1 tract arising the origin of the ophthalmic artery (Oph A). The right carotid injection revealed the existence of an abnormal vessel branching from the intradural origin of the carotid siphon (Figs. 1a and 1b). This vessel terminated in the region of the AcoA. It followed an oblique path superiorly and medially which was concave toward the right and superiorly. On the oblique views, part of its course projected below the image of the optic canal. The aneurysm appeared to be situated on this vessel proximal to the infered site of the AcoA.

**Figure 2:**
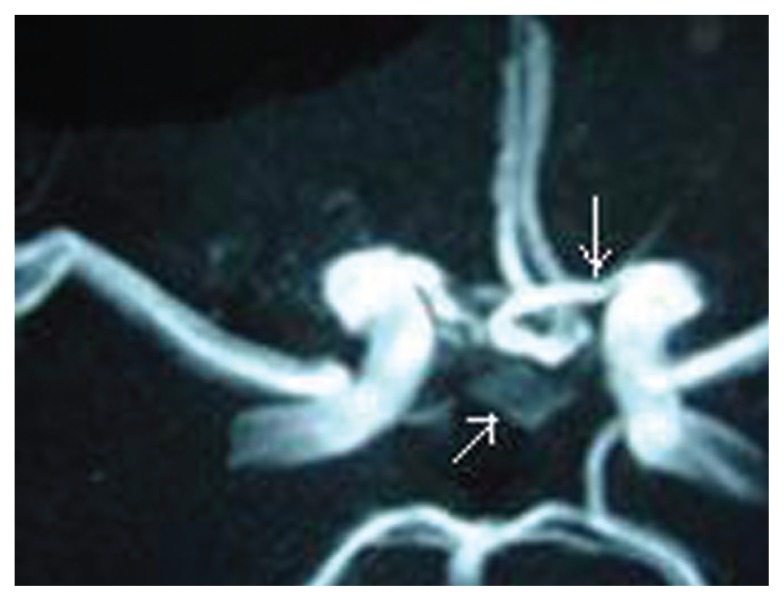
Postoperative magnetic resonance angiography showing clipped an AcoA aneurysm. Arrowheads, anomalous infraoptic proximal tract (A1) of the anterior cerebral artery and documented the successful exclusion of aneurysm.

Operation: surgery was performed at the 6th day after hemorrhage. A right pterional craniotomy was performed. After the carotid and chiesmatic cisterns were opened, a low bifurcation of the ICA was observed. The anomalous artery was identified as it appeared from beneath the optic nevre and was followed to the aneurysm after the proximal A1 segment coursed under the ipsilateral optic nerve and the distal A1 segment ran anterior to the chiasm. The anterior communicating artery aneurysm was clipped. The post operative course was unventful, with complete recovery. Postoperative CT scan was normal. The anomalous infraoptic proximal tract of the anterior cerebral artery was documented by magnetic resonance angiography (Fig. 2) and three-dimensional CT angiogaphy after surgery. Follow-up documented the successful exclusion of aneurysm.

## Discussion

Aneurysms of the complex of the ACA are frequently associated with anatomic variations of the circle of Willis (Chioffi et al. 1997; Kessler, 1979; Kwak et al. 1980; Lasjaunias et al. 2001). Although hypoplasia of the A1 tract and presence of three distal ACAs can be clearly recognized in preoperative angiograms other anatomic variations, such a fenestrations of the AComA are generally discovered only during surgery (Dawson, 1958; Gomes et al. 1986; Lasjaunias et al. 2001).

The presence of an infraoptic cource of the A1 tract is an extremely rare anomaly (Bassett, 1949; Besson et al. 1980; Bollar et al. 1988; Brismar et al. 1977; Chioffi et al. 1997; Dawson, 1958; Kwak et al. 1980; Lasjaunias et al. 2001; Mercier et al. 1989; Yaşargil, 1984). This anomaly represents a maldevelopment in the embryogenesis of the anterior circle of, Willis, resulting from the persistence of the primitive prechiasmal arterial anastomosis or an error in the development of the definitive ophthalmic artery. Aplasia or hypoplasia of the A1 segment, fenestration or duplication of the AComA, and the presence of three distal ACAs are frequently reported anomalies (Chioffi et al. 1997; Dawson, 1958; Gomes et al. 1986; Kwak et al. 1980). Among patients with A ComA aneurysms hypoplasia of the A1 segment was reported for 80% of patients, according to Yaşargil, 1984 (Yaşargil, 1984), or 35% of patients according to Chioffi et al. (Chioffi et al. 1997), fenestration and duplication of the ACom A were reported for 4.4% (Chioffi et al. 1997) and 22.4% (Yaşargil, 1984) of cases, and three distal ACAs were documented for 3.7% of the patients in the series of Chioffi et al. (Chioffi et al. 1997) and 9.6% of the surgical observations of Yaşargil (Yaşargil, 1984). An infraoptic course of the proximal anterior cerebral artery is a rare anomaly that has been reported in 32 cases to date, often in association with cerebral aneurysms (Bassett, 1949; Besson et al. 1980; Bollar et al. 1988; Brismar et al. 1977; Chioffi et al. 1997; Dawson, 1958; Gomes et al. 1986; Kessler, 1979; Kwak et al. 1980; Lasjaunias et al. 2001; Mercier et al. 1989; Yaşargil, 1984).

The ACA normally courses from the ICA bifurcation medially and often somewhat anteriorly, toward the interhemispheric fissure, passing over the optic nerves and chiasm (supraoptic course). In all cases observed the infraoptic course of the A1 tract is associated with a low bifurcation of the ICA, at the level of Oph A( just as it becomes intradural) or above (Chioffi et al. 1997; Lasjaunias et al. 2001). In our case and in others, the origin of the anomalous A1 tract is in common with that of the OphA, and this may have a precise embryological significance.

Patients with a supraoptic course of the proximal tract of the ACA have exhibited a variety of symptoms, mostly resulting from ruptured associated AComA aneurysms and occasionally resulting from compression of the optic nevre or chiasm by the anomalous vessel. In our case, an extremely rare variation of the proximal tract of the ACA, i.e. an infraoptic course of the proximal precommunicating tract (A1) under the optic nerve, with the distal A1 tract anterior to the chiasm and positioned between the optic nerves, is presented.

## Conclusion

The anomalous infraoptic course of the proximal anterior cerebral artery was associated with a low bifurcation of the ipsilateral internal carotid artery in this patient. The anterior cerebral a. arises a few millimetres above the emergency of the internal carotid from the cavernous sinus, at the usual level of the ophthalmic a. It travels medially, beneath the optic n., and then describes a curve with a superolateral concavity to arrive at the anterior communicating a. The recognition of this variant is very important for allows optimal surgical planning and forestalls misinterpreation of the individual cerebral vascular anatomic features and possible complications during surgery.

## Figures and Tables

**Figure 1a:**
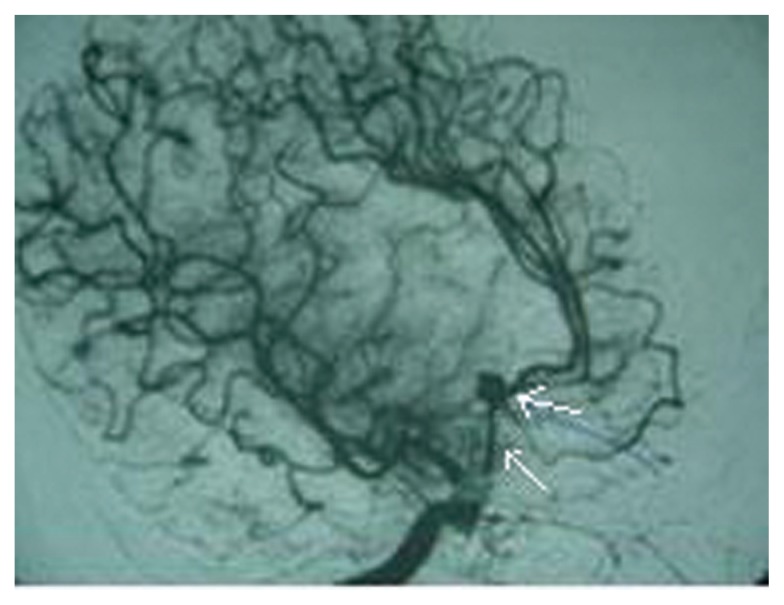
Lateral view from right carotid artery angiography, showing an AcomA aneurysm and a low bifurcation of the right internal carotid artery (ICA), an anomalous A1 tract arising the origin of the ophthalmic artery (Oph).

**Figure 1b:**
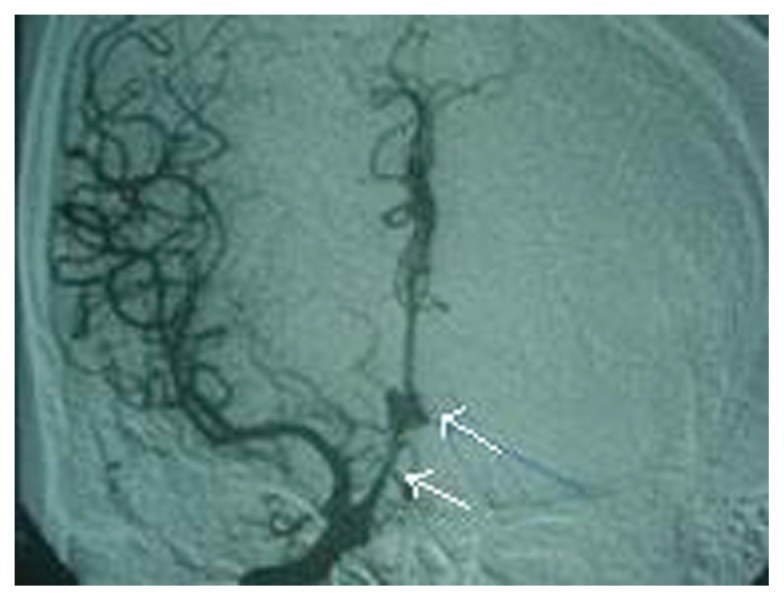
Anteroposterior view from right carotid artery angiography, showing an AcomA aneurysm. Arrowheads, anomalous A1 tract and AcomA aneurysm.

## Abbreviations

A1: proximal tract of the anterior cerebral artery
A2: distal tract of the anterior cerebral artery
AcomA: anterior communicating artery
CT: Computed Tomography
ICA: internal carotid artery
Oph A: ophthalmic artery
